# The Effect of the DemensiaKITA Mobile Health App Intervention on Knowledge, Attitude, Practice, and Burden Level of Dementia Caregivers in Kuala Lumpur and Selangor, Malaysia: Protocol for a Nonrandomized Controlled Trial

**DOI:** 10.2196/83699

**Published:** 2026-02-25

**Authors:** Nurulizzah Mahfar, Nik Nairan Abdullah, Dalila Roslan, Khalid Ibrahim, Ungku Ahmad Ameen Ungku Mohd Zam, Noraliza Noordin Merican, Xin Wee Chen, Mariam Mohamad

**Affiliations:** 1 Public Health Medicine Department, Faculty of Medicine, Universiti Teknologi MARA, Sg Buloh Campus, Selangor Branch, Sungai Buloh, 47000, Malaysia, +60361267214; 2 Geriatric Unit, Medical Department, Hospital Tengku Ampuan Rahimah, Klang, Malaysia; 3 Elderly Health Sector, Family Health Development Division, Ministry of Health, Putrajaya, Malaysia

**Keywords:** dementia, caregivers, caregiver burden, health knowledge, attitudes, practice, mobile apps, nonrandomized controlled trial

## Abstract

**Background:**

Dementia is a growing public health concern, disproportionately affecting low- and middle-income countries. Caregivers of people living with dementia often face significant physical, psychological, social, and financial burdens, with high prevalence rates of caregiver strain in Malaysia. Mobile health (mHealth) apps have demonstrated potential to enhance caregivers’ knowledge, attitudes, and practices (KAP) and to reduce burden. However, few culturally tailored solutions exist for Malaysia. The *DemensiaKITA* app was developed to provide locally relevant information, support services, and stress management tools for dementia caregivers.

**Objective:**

The purpose of this single-blinded, nonrandomized controlled trial (NRCT) is to evaluate the effectiveness of the *DemensiaKITA* mHealth app in improving caregivers’ KAP and reducing caregiver burden in Selangor and Kuala Lumpur, Malaysia.

**Methods:**

This research will be conducted in 2 phases. Phase 1 involves the adaptation, translation, and validation of 4 instruments: Dementia Knowledge Assessment Tool Version 2, Dementia Attitudes Scale, Caregiver Task Inventory—25 items, and Short-form Zarit Burden Interview—12 items. Content, face, and construct validity, along with reliability (Cronbach α and test-retest intraclass correlation), will be established. Phase 2 is a single-blinded NRCT conducted in 4 public hospitals. A total of 100 caregivers will be recruited. Two intervention hospitals will receive the *DemensiaKITA* app, while 2 control hospitals will receive usual care (Ministry of Health dementia leaflets and video). The primary outcomes (KAP) and secondary outcome (burden level) will be assessed at baseline, 1 month, and 3 months using the validated questionnaires on an intention-to-treat basis. Data will be analyzed with descriptive statistics, chi-square and *t* tests, and generalized estimating equations.

**Results:**

Recruitment and baseline data collection are underway. Recruitment for Phase 1 (instrument validation) occurred from December 2024 until July 2025. It is expected to produce valid and reliable Malay versions of KAP and burden instruments. Recruitment for the Phase 2 NRCT will occur from August 2025 until February 2026. The first follow-up data collection for Phase 2 will occur 1 month after baseline, and the second and final follow-up will occur at 3 months. Data analysis has not yet begun. Phase 2 is expected to show significant improvements in dementia KAP and reduced caregiver burden in the intervention group compared to controls. This study was conducted without any specific external financial support or grants from any public, commercial, or not-for-profit funding agencies. The study findings are expected to be published in December 2026.

**Conclusions:**

This article describes the protocol for a single-blinded NRCT examining a novel mHealth intervention. The *DemensiaKITA* app has the potential to empower caregivers, enhance dementia care practices, and alleviate caregiver burden in Malaysia and may serve as a model for other low- and middle-income countries globally.

## Introduction

Dementia is a progressive syndrome associated with cognitive decline and behavioral and psychological symptoms, requiring substantial support from caregivers [1]. In 2018, an estimated 50 million people were living with dementia globally, with 60% residing in low- and middle-income countries [2]. In Malaysia, the prevalence of dementia among older adults is 8.5% [1,3].

Caregivers often experience significant physical, emotional, social, and financial strain [1,2,4,5], exacerbated in low-income countries where formal support services for people living with dementia remain limited [3,6]. Caregiver burden is highly prevalent, with global estimates at 49.26% and Malaysian estimates reaching 69.4% [7,8]. Additionally, a longitudinal study in Sydney, Australia, demonstrated an increase in caregiver burden from 47.4% at baseline to 56.8% after 3 years [9].

Research consistently shows substantial gaps in dementia knowledge and variable attitudes among caregivers. A survey in China reported low knowledge despite moderate attitudes [10], while a study in India found that many caregivers had only average or below-average knowledge, low to moderate attitudes, and predominantly poor caregiving practices. Evidence indicates these gaps can be improved through targeted interventions. For example, a randomized controlled trial in eastern Taiwan showed that mobile eLearning significantly enhanced dementia knowledge, attitudes, and care competency among home care workers [11]. Positive attitudes have also been associated with better caregiving practices, highlighting the value of interventions that strengthen both knowledge and attitudes through communication training, safe care techniques, behavioral management strategies, psychological support, and information on available services [10].

The rapid expansion of internet access has enabled the use of mobile health (mHealth) apps to support caregivers [3]. Prior studies demonstrate that mHealth tools can provide health information, connect users with local services, and reduce caregiver burden [5,12-14]. However, most dementia-focused apps are developed in high-income countries and predominantly in English, limiting their applicability in diverse cultural contexts [12,15,16]. More rigorous evaluations are also needed to determine how mHealth interventions influence caregivers’ knowledge, attitudes, and practices (KAP) and burden level [10,17-19].

*DemensiaKITA* is a Malaysian mHealth prototype developed to address this gap. The app provides information on dementia, caregiving strategies, management of behavioral symptoms, stress-reduction techniques, and access to local health and community resources. Its content is culturally tailored and delivered in Malay, developed collaboratively by dementia care experts and technology researchers [12]. However, its effectiveness in improving caregivers’ KAP and reducing burden has not yet been evaluated.

This study aims to (1) adapt, translate, and validate Malay-language questionnaires assessing dementia caregivers’ KAP and burden, and (2) evaluate the effectiveness of the *DemensiaKITA* mHealth app in improving KAP and reducing caregiver burden among dementia caregivers in Selangor and Kuala Lumpur through a nonrandomized controlled trial.

## Methods

### Study Design

This study consists of 2 sequential phases: (1) adaptation, translation, and validation of KAP and burden-related instruments, and (2) evaluation of the *DemensiaKITA* mHealth intervention via a single-blinded NRCT. The NRCT design was selected due to operational and ethical constraints within participating hospitals. Caregivers are tied to specific clinics based on people living with dementia follow-up, and redistributing them across hospitals for randomization would disrupt continuity of care. Cluster randomization was not feasible due to small eligible populations within each site. Individual randomization within the same clinic would introduce high contamination risks, as caregivers frequently interact and could share intervention materials.

To mitigate potential biases inherent in nonrandom allocation, the study incorporates several methodological safeguards: (1) standardized inclusion and exclusion criteria across all sites; (2) selection of hospitals with comparable geriatric services; (3) baseline comparison of groups; and (4) longitudinal analyses using generalized estimating equations (GEE) to adjust for baseline imbalances and repeated measurements [20,21]. This pragmatic design reflects real-world implementation conditions while preserving internal validity.

### Study Population

The target population is dementia caregivers in Malaysia, while the accessible study population comprises primary caregivers of people living with dementia aged 18 years and older in Selangor. The sampling population is caregivers of registered people living with dementia attending the study sites from December 2024 to February 2026. The sampling unit is a consenting primary caregiver providing direct care to registered people living with dementia.

### Phase 1: Instrument Adaptation, Translation, and Validation

#### Overview

Phase 1 involves adapting, translating, and validating 4 instruments into the Malay language: Dementia Knowledge Assessment Tool Version 2 (DKAT-2) [22], Dementia Attitudes Scale (DAS) [23], Caregiver Task Inventory—25 items (CTI-25) [24,25], and the Short-form Zarit Burden Interview—12 items (ZBI-12) [26-28]. It is scheduled from December 2024 to July 2025.

**Figure 1.**
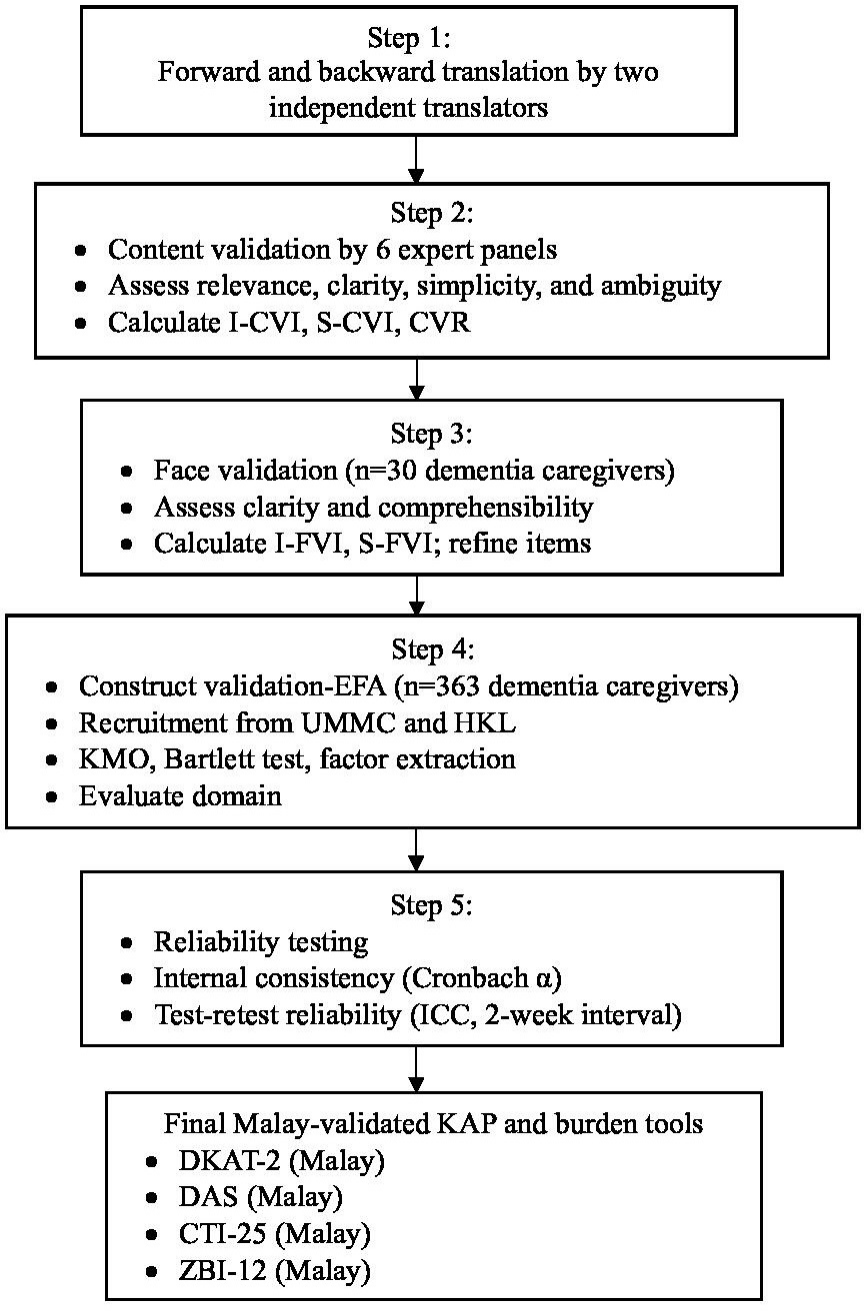
Study flow chart for Phase 1: adaptation, translation, and validation. CTI-25: Caregiver Task Inventory—25 items; CVR: content validity ratio; DAS: Dementia Attitudes Scale; DKAT-2: Dementia Knowledge Assessment Tool Version 2; EFA: exploratory factor analysis; HKL: Hospital Kuala Lumpur; ICC: intraclass correlation coefficient; I-CVI: item-level content validity index; I-FVI: item-level face validity index; KAP: knowledge, attitudes, and practices; KMO: Kaiser-Meyer-Olkin; S-CVI: scale-level content validity index; S-FVI: scale-level face validity index; UMMC: Universiti Malaya Medical Center; ZBI-12: Short-form Zarit Burden Interview—12 items.

There will be forward and backward translations of the questionnaires by two independent translators. Two independent translators will translate the original questionnaires into Malay and then back into the original language. This process helps identify and rectify any discrepancies or inconsistencies in meaning. The drafted Malay versions of the KAP questionnaires will be rigorously validated, encompassing content validation, face validation, construct validation, and an assessment of reliability (Figure 1). Eligibility criteria for Phase 1 are given in Textbox 1.

Textbox 1. **The inclusion and exclusion criteria for Phase 1.**

**Inclusion criteria**

1. A primary caregiver of people living with dementia (a person who has helped in the personal care of a person living with dementia for 1 mo or more during the last 12 mo)
2. Aged 18 years or older
3. Cares for older adult aged 60 years or older with any form and severity of dementia
4. Informal and unpaid caregivers
5. Minimum of at least 4 h of care per day

**Exclusion criteria**

1. A person who is unable to communicate independently in Bahasa Malaysia
2. Domestic helper

#### Content Validity

Content validity will be evaluated by a panel of 6 experts comprising public health physicians, geriatricians, and 1 informal caregiver of people living with dementia who will serve as an experiential expert to ensure contextual and practical relevance for the target population. Each panel member will independently assess all the items for relevance, clarity, simplicity, and the absence of ambiguity, following established content-validity assessment procedures [29-32]. The ratings provided will be used to compute item-level and scale-level content validity indices (CVIs).

#### Face Validity

Face validity will be examined among 30 dementia caregivers who will assess the clarity and comprehensibility of each item. Their feedback will be used to calculate the face validity index in accordance with recommended guidelines [33-35]. This process will ensure that the instrument is clear and understandable to the target population.

#### Construct Validity

Construct validity will be assessed using exploratory factor analysis (EFA). Based on the recommended participant-to-item ratio of at least 5:1, a minimum of 330 participants is required, with an additional 10% to account for potential attrition, yielding a total target sample of 363 participants. EFA will be performed to evaluate the underlying factor structure and to determine whether the items load appropriately onto the intended domains [36,37]. Participants will be recruited through convenience sampling from the Geriatric Clinic at Universiti Malaya Medical Center (UMMC), Kuala Lumpur, and Hospital Kuala Lumpur. This sample size is expected to provide adequate power and stability for robust factor extraction and model interpretation.

#### Reliability

Reliability will be assessed through internal consistency and test-retest procedures. Internal consistency will be evaluated using Cronbach α for each domain and for the overall scale. Test-retest reliability will be examined over a 2-week interval using intraclass correlation coefficient, following established guidelines for reliability assessment [38-41]. This combined approach will ensure stability and consistency of the instrument across time.

### Phase 2: Effectiveness of the *DemensiaKITA* mHealth App

#### Overview

**Figure 2.**
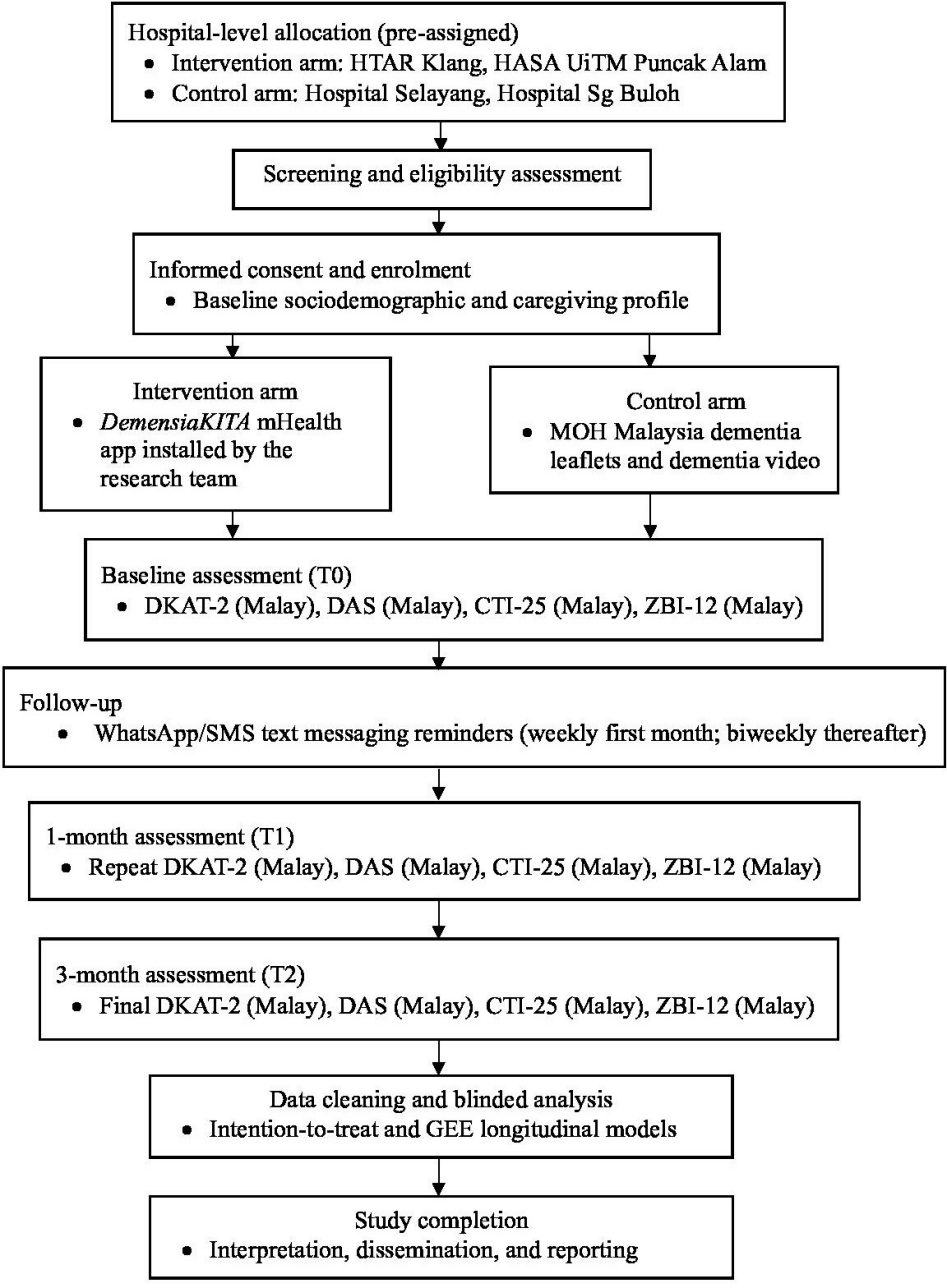
Study flowchart for Phase 2: effectiveness trial (nonrandomized controlled trial). CTI-25: Caregiver Task Inventory—25 items; DAS: Dementia Attitude Scale; DKAT-2: Dementia Knowledge Assessment Tool Version 2; GEE: generalized estimating equations; HASA: Hospital Al-Sultan Abdullah; HTAR: Hospital Tengku Ampuan Rahimah; MOH: Ministry of Health; UiTM: Universiti Teknologi MARA; ZBI-12: Short-form Zarit Burden Interview—12 items.

In Phase 2, the research assesses the effectiveness of the *DemensiaKITA* mHealth app intervention among dementia caregivers in Selangor, Malaysia. This phase uses an NRCT with 2 parallel arms (intervention and control) and a 1:1 allocation ratio. The study includes 3 assessment points: baseline (T0), 1 month (T1), and 3 months (T2). The intervention period spans from August 2025 to February 2026. Participants will be blinded to group allocation (single-blinded). Hospital-level allocation is applied to minimize contamination (Figure 2). Eligibility criteria for Phase 2 are given in Textbox 2.

Textbox 2. **Inclusion and exclusion criteria for Phase 2.**

**Inclusion criteria**

1. Primary caregiver of people living with dementia who has provided personal care for 1 month or more
2. Aged 18 years or older
3. Caring for an older adult aged 60 years or older with any type of dementia
4. Informal and unpaid caregiver
5. Minimum of at least 4 hours of care per day
6. Possesses an operational Android smartphone with mobile data
7. Able to use basic smartphone functions and apps

**Exclusion criteria**

1. Unable to communicate independently in Bahasa Malaysia
2. Not an Android smartphone user
3. Employed domestic helper

#### Study Settings

The trial will be conducted in the geriatric clinics of 4 public hospitals in Selangor, Malaysia: (1) Hospital Tengku Ampuan Rahimah, Klang; (2) Hospital Sungai Buloh; (3) Hospital Selayang; (4) Hospital Al-Sultan Abdullah, Universiti Teknologi MARA (UiTM) Puncak Alam. These settings were chosen due to their established geriatric subspecialty services and continuous caseload of dementia caregivers. To prevent contamination, hospitals were allocated based on geographic distribution and patient-flow patterns—Intervention arm: Hospital Tengku Ampuan Rahimah, Klang, and Hospital Al-Sultan Abdullah, UiTM Puncak Alam; Control arm: Hospital Selayang and Hospital Sungai Buloh (Figure 2).

Because follow-up care rarely overlaps across hospitals, this site-based allocation minimizes cross-exposure. The *DemensiaKITA* app was installed only by the research team on participants’ smartphones in the intervention arm. Caregivers were advised not to share the app or its contents during the study period.

#### Recruitment Strategy

Participants will be identified using purposive sampling among caregivers of registered people living with dementia attending the 4 participating geriatric clinics. Screening and recruitment will be conducted by the research team. Eligible caregivers will receive a full explanation of the study objectives, procedures, confidentiality safeguards, and follow-up requirements.

#### Allocation and Blinding

Because hospitals were preassigned to their respective study arms, no individual-level randomization will be implemented. The study will be conducted as a single-blinded trial whereby participants will not be informed of their allocation to either the intervention or control group and will instead be informed that they will receive “health education material,” as stated in the patient information sheet. In addition, researchers responsible for data analysis will remain blinded to group assignment until all data have been cleaned and finalized to minimize analysis-related bias.

#### Intervention (Intervention Arm)

Participants assigned to the intervention hospitals will receive the *DemensiaKITA* mHealth app. The app will be installed directly by the research team to prevent unauthorized sharing. Participants may access the app freely during the study period.

### DemensiaKITA Mobile Health App Intervention

#### Overview

**Figure 3.**
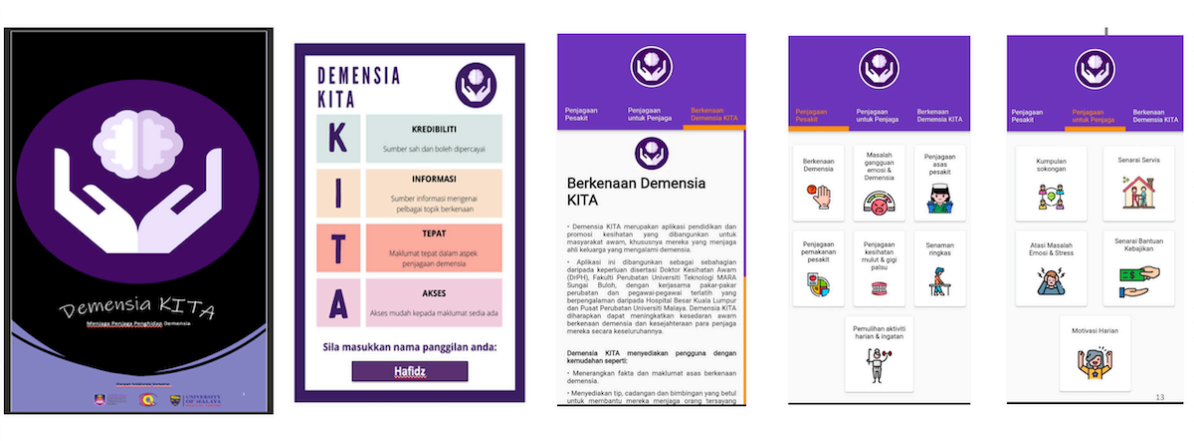
Features of *DemensiaKITA* mobile health app.

The *DemensiaKITA* app is an offline mobile app prototype developed by a team of researchers from Universiti Teknologi MARA, Malaysia, and the Canadian University of Dubai (Figure 3). The app has been granted copyright protection under Universiti Teknologi MARA. It provides essential information on dementia and dementia care, facilitates easy access to nearby health services, optimizes the use of available local resources, and enhances caregiving skills. The app features appropriate and relevant content tailored for the Malaysian context, using the national language (Bahasa Malaysia) as the interface, specifically designed for dementia caregivers in Malaysia (Figure 3 and Textboxes3  4) [12].

Textbox 3. **Additional features of the *DemensiaKITA* app (support functions to reduce caregiver burden).**

- Stress and emotional management: Resources to help caregivers cope with stress and emotional challenges, potentially reducing their emotional burden.
- Daily motivation: Encouragement and inspiration to support caregivers in their daily routines, potentially reducing their emotional and social burdens.
- Support group: Access to online and in-person support groups, helping caregivers connect with others and reduce feelings of isolation, potentially reducing emotional and social burdens.
- List of available services (including health services, nongovernment organizations, daycare, and nursing home): A comprehensive list of available services, enabling caregivers to find the support they need and potentially reduce their physical burden by facilitating access to care.
- List of available welfare aid: Details on available financial assistance and welfare programs to help alleviate the financial burden of caregiving.

Textbox 4. **Basic features of the *DemensiaKITA* app (information and guidance on dementia care).**

The *DemensiaKITA* app aims to improve the caregivers’ knowledge, attitudes, and practices in taking care of people living with dementia. It provides information and guidance on various aspects of dementia care:

- Regarding dementia
- Emotional disturbance of people living with dementia
- Basic care of people living with dementia
- Dietary care of people living with dementia
- Oral exercise for people living with dementia
- Basic exercise for people living with dementia
- Physical and memory rehabilitation

#### Engagement and Adherence Monitoring

To promote engagement with the *DemensiaKITA* app, caregivers in the intervention group will receive a brief, standardized orientation on the app features at enrollment. Thereafter, the research team will send scheduled reminders via WhatsApp or SMS text messaging to encourage continued use of the app and completion of follow-up assessments. Reminders will be sent once weekly during the first month and every 2 weeks thereafter until the 3-month follow-up.

#### Comparator (Control Arm)

Caregivers in the control arm will receive standard Ministry of Health (MOH) dementia educational materials, which are the dementia leaflets and video. These materials will be explained in detail during the baseline session to ensure consistent educational exposure and engagement comparable to that provided in the intervention arm [42].

#### Dementia Leaflets and Video

**Figure 4.**
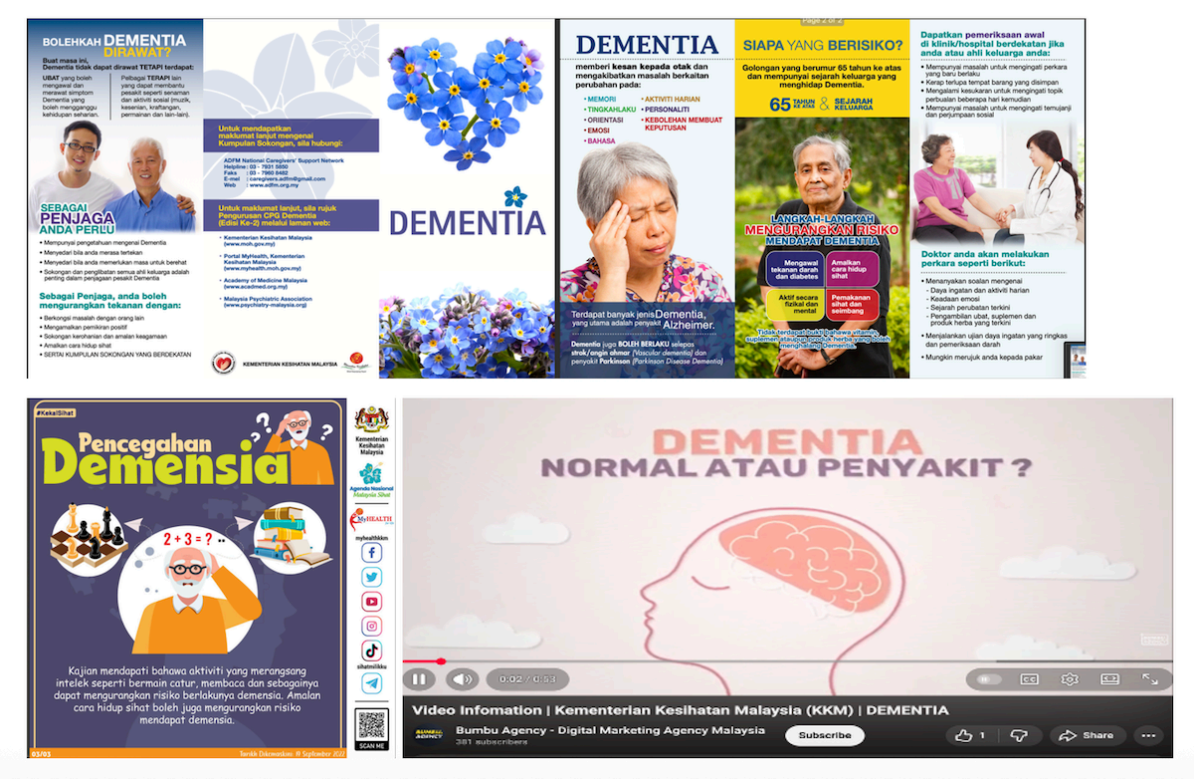
Ministry of Health Malaysia leaflets and video.

The dementia leaflets and video provided by the MOH Malaysia offer essential information on the nature of dementia, its common symptoms, and the groups at higher risk (Figure 4). They emphasize the importance of early detection and timely diagnosis, describe the available assessments conducted in clinics and hospitals, and outline practical steps to help maintain cognitive and physical well-being. The materials also highlight key measures that may reduce dementia risk, including mentally stimulating activities, regular exercise, healthy lifestyle practices, and effective management of chronic illnesses. In addition, they provide guidance on daily care, safety, behavioral management, and available support resources to assist people living with dementia and their caregivers [42].

### Data Collection Method and Study Tool

Data will be collected using a validated self-administered questionnaire at 3 time points: baseline, 1 month postintervention, and 3 months postintervention. The study tool is a Malay-translated and validated questionnaire assessing KAP and burden levels. Participants in the intervention group will have access to the mobile app 2 weeks before the study starts to get familiar with it.

Before the study begins, all participants will be informed of the study objectives, procedures, potential risks, and benefits and will provide informed consent. Participation is voluntary, and participants may withdraw at any time without penalty. To help keep participants engaged, reminders will be sent via WhatsApp or email throughout the study. These reminders will also check on app use for the intervention group and encourage continued participation until 3 months postintervention.

### Sample Size Calculation

The sample size for Phase 2 was calculated for comparison of 2 independent means (intervention vs control) assuming equal group sizes, a 2-sided test with a significance level of *α*=.05, and power (1−β)=0.80. The calculation followed the method described by Rosner [43] and Turnbull et al [44]. The required sample size per group was obtained using the formula:


$$n=\frac{(\sigma_{1}^{2}+\sigma_{2}^{2})(Z_{1-\alpha /2}+Z_{1-\beta }{)}^{2}}{\Delta^{2}}$$

where $\sigma_{1}$ and $\sigma_{2}$ are the SDs in the 2 groups, $Z_{1-\alpha /2}$ is the standard normal deviate at the 5% (1.96) significance level, $Z_{1-\beta }$ is the standard normal deviate for 80% (0.84) power, and Δ is the expected mean difference between the groups.

Based on Turnbull et al [44], an expected mean difference in knowledge scores of 1.69 was used, with SDs of 2.53 and 2.76, respectively. Substituting these values:


$$n=\frac{(2.53^{2}+2.76^{2})(1.96+0.842{)}^{2}}{1.69^{2}}\approx 38.5$$

Thus, approximately 39 participants are required per group. To account for an anticipated 30% attrition rate, the sample size was inflated:


$$n_{\text{adjusted}}=38.5\times 1.30\approx 50$$

Accordingly, 50 participants will be recruited into each arm (intervention and control), giving a total sample size of 100 caregivers.

### Study Outcome

The outcomes of this study will be evaluated using validated instruments measuring caregivers’ KAP and burden level in relation to caring for people living with dementia.

#### Knowledge

Caregiver knowledge will be assessed using the DKAT-2, developed by Toye and colleagues [22] at Curtin University. The DKAT-2 consists of 21 items with Yes/No/Don’t Know response options and measures understanding of the dementia trajectory and evidence-based care approaches. Each item is scored as correct or incorrect, with “Don’t Know” categorized as incorrect. Total scores reflect overall knowledge, with higher scores indicating greater understanding of dementia care. The DKAT-2 demonstrates strong psychometric performance, including high internal consistency (Cronbach *α*=0.79), supporting its use in evaluating caregiver education and guiding patient-centered interventions [22].

#### Attitude

Attitudes toward dementia will be measured using the DAS, developed by O’Connor and McFadden [23]. The DAS comprises 20 items rated on a 7-point Likert scale (1=strongly disagree to 7=strongly agree), capturing cognitive, emotional, and behavioral components of attitudes toward dementia. Higher scores denote more positive attitudes. The scale has demonstrated excellent reliability (Cronbach *α*=.83‐.85) and a stable 2-factor structure confirmed through exploratory and confirmatory factor analyses [23]. Its broad conceptual coverage makes it suitable for evaluating the impact of educational and psychosocial interventions.

#### Practice

Caregiving practices will be assessed using the CTI-25. The CTI-25 is a shortened and refined version of the original 45-item instrument developed by Clark and Rakowski in 1983 [24], with improvements introduced by Lee and Mok [25] to enhance clarity and reduce redundancy. The instrument contains 25 items across 5 domains: learning to cope with a new role; providing care based on the care recipient’s needs; managing one’s emotional needs; appraising supportive resources; and balancing personal and caregiving demands.

Items are rated on a 3-point Likert scale (0‐2), with higher scores reflecting greater caregiving difficulty. The CTI-25 demonstrates strong psychometric properties, including high internal consistency (*α*=.93) and construct validity supported by confirmatory factor analysis [25].

#### Burden Level

Caregiver burden will be measured using the ZBI-12, a validated abbreviated version of the original 22-item ZBI developed by Zarit in 1980 [26-28]. The ZBI-12, translated into Malay in 2023, evaluates key burden domains including privacy, social life impact, emotional strain, health effects, and time-related pressures. The shorter format enables rapid screening and ongoing monitoring while retaining strong psychometric performance. Total scores are categorized as: 0‐10 (no to mild burden); 11‐20 (mild to moderate burden); and 20 or more (high burden) [26-28].

### Harms and Safety Monitoring

This study evaluates an educational mHealth app and standard health education materials, which are considered minimal-risk behavioral interventions. No physical risks are anticipated. Harms will therefore be defined as any self-reported serious psychological distress or any adverse event that participants or clinicians consider to be related to use of the app or study procedures. At each follow-up contact, participants will be asked whether they experienced any problems or distress related to the intervention or study participation. Participants may discontinue app use or withdraw from the study at any time on request, without consequences for their routine care. Any serious adverse events judged to be related to the study will be reported to the relevant ethics committees in accordance with institutional policies.

### Ethical Considerations

#### Ethical Approval

This study received ethical approval from multiple institutional review boards. Approval was first obtained from the Faculty Ethics Research Committee, UiTM on November 7, 2024 (REC/11/2024 [PG/MR/549]). The study was subsequently approved by the Medical Research and Ethics Committee, Ministry of Health Malaysia, on December 16, 2024 (24‐03494-W6I [IIR]), in accordance with the ethical standards of the National Institutes of Health Malaysia. Additional approval was granted by the Medical Research Ethics Committee, UMMC, on May 21, 2025 (2025122‐14637).

Operational approvals were obtained from the Directors of Hospital Kuala Lumpur and UMMC for Phase 1 and from the Director of Selangor State Health Department and the Directors of the 4 participating hospitals for Phase 2. Permission to use all study instruments was formally granted by the respective copyright holders and instrument developers.

Before the study begins, all participants will be informed of the study objectives, procedures, and potential risks and benefits and will provide informed consent. Written informed consent will be obtained prior to enrollment. No financial or material compensation will be provided to participants for their participation in this study.

#### Data Management and Security

Participant data will be obtained through both hard-copy questionnaires and electronic forms, then entered into a password-protected database accessible only to authorized research staff. Double data entry and range checks will be performed for key variables to minimize errors. All electronic data will be stored on secure institutional servers with regular backup. Identifiers (names, phone numbers, and identification number) will be stored separately from research data and linked only through a unique study identification code. The *DemensiaKITA* app does not collect or transmit any identifiable health information; it functions as an information and support tool installed locally on participants’ smartphones.

#### Confidentiality

All personal information obtained from participants will be treated as confidential and used solely for research purposes. Identifiable data will not be included in publications or presentations. Hard-copy documents will be stored in locked cabinets within restricted-access offices. Electronic data will be deidentified for analysis, and only the principal investigator and authorized team members will have access to the code key. Research procedures will comply with the Malaysian Personal Data Protection Act and institutional data protection policies.

#### Data Sharing and Availability

Deidentified individual participant data underlying the main trial results may be made available upon reasonable request to the corresponding author after publication of the primary findings, subject to institutional data-sharing policies and approval by the ethics committees.

### Dissemination Policy

The results of this trial will be disseminated through peer-reviewed publications, conference presentations, and updates to the ClinicalTrials.gov registry record. A lay summary of the main findings will be prepared in Bahasa Malaysia and made available to participating clinics and interested caregivers.

## Results

### Phase 1: Questionnaire Adaptation, Translation, and Validation

This phase focuses on adapting, translating, and validating the questionnaires on KAP and burden level. The following outcomes are anticipated.

#### Content Validation

Content validation will be assessed using the CVI and content validity ratio. To ensure rigorous content validation, an item-level CVI of 0.79 or higher is required for item retention, while a scale-level CVI of 0.80 or higher is necessary to establish excellent content validity for the overall scale. A minimum content validity ratio of 0.99 is established based on expert consultation. This rigorous approach ensures that only the most relevant and important items are retained for subsequent phases of instrument development [29-31,34].

#### Face Validation

Face validation will be assessed by calculating the face validity index (FVI) to quantify the degree of agreement among respondents regarding the clarity and comprehensibility of the questionnaire items. A sample of 30 respondents was used for this process. Items with an item-level FVI and scale-level FVI of 0.80 or higher were retained. This threshold aligns with recommendations for establishing strong face validity [33-35].

#### Construct Validation

Construct validation will be conducted using EFA to identify the underlying factor structure of the questionnaire. This will confirm that the items are effectively measuring the intended concepts. Additionally, correlational analysis will examine the relationships between different scales within the questionnaire to assess convergent and discriminant validity [34,36,45].

#### Reliability

To ensure the questionnaire’s consistency, 2 key aspects of reliability will be evaluated. First, internal consistency will be assessed using Cronbach α, which measures how well the items within the questionnaire work together to measure the same concept. A Cronbach α of .70 or higher will be considered acceptable. Second, test-retest reliability will be assessed using the intraclass correlation coefficient. This determines the stability of the questionnaire’s results over time, with an acceptable value of 0.75 or higher [34,39,41]. This 2-pronged approach ensures the questionnaire produces reliable and consistent results.

### Phase 2: Intervention Effectiveness and Data Analysis

This phase focuses on evaluating the effectiveness of the *DemensiaKITA* mHealth app compared to the usual care.

#### Inferential and Longitudinal Analysis

All analyses will be conducted using a 2-sided significance level of *α*=.05. Descriptive statistics will summarize sociodemographic and caregiving characteristics by study arm [46]. Categorical variables will be presented as frequencies and percentages, and continuous variables as means and SDs or medians and IQRs, as appropriate. Baseline comparability between the intervention and control groups will be examined using chi-square tests for categorical variables and independent *t* tests (or nonparametric equivalents) for continuous variables [46,47].

The primary effectiveness analysis will use generalized estimating equations models to compare changes in knowledge, attitude, practice, and burden scores between the intervention and control groups over time (baseline, 1 mo, and 3 mo). For each outcome, a GEE model with an appropriate link function and working correlation structure will be fitted, including fixed effects for group (intervention vs control), time, and the group-by-time interaction. The group-by-time interaction term will be interpreted as the main estimate of the intervention effect. To account for potential confounding due to nonrandom allocation, the models will adjust for prespecified baseline covariates (age, sex, and relationship with the people living with dementia, caregiving duration, and baseline score of the corresponding outcome). Robust standard errors will be reported [21,48].

Effect sizes (Cohen *d* for between-group differences in mean change) will be calculated to quantify the magnitude of the intervention effect, in addition to *P* values and 95% CIs [21,48].

#### Analysis Population, Missing Data, and Sensitivity Analyses

The primary analysis will follow the intention-to-treat principle, in which all participants will be analyzed in the groups to which their hospital was allocated, regardless of their level of app use, exposure to the leaflets, or subsequent adherence. Every effort will be made to obtain outcome data at 1 and 3 months for all enrolled caregivers, including those who discontinue or reduce use of the intervention [49].

Missing outcome data will initially be handled within the GEE framework, which uses all available repeated measurements under a missing-at-random assumption and provides robust standard errors. Patterns and reasons for missing data will be described. If more than a small proportion of outcome data (>20%) is missing, multiple imputation by chained equations will be considered as a sensitivity analysis, with covariates and outcome scores imputed under the missing-at-random assumption. Results from imputed and complete-case analyses will be compared to assess the robustness of the findings [21].

This study was conducted without any specific external financial support or grants from any public, commercial, or not-for-profit funding agencies. Recruitment and baseline data collection are currently underway at the time of manuscript submission (phase 1: 210 participants, phase 2 not yet started at the time of submission). The study findings are expected to be published in December 2026.

## Discussion

### Principal Findings

Caring for people living with dementia in low-income countries like Malaysia presents significant challenges for caregivers [2,8]. They often experience physical and emotional exhaustion from providing constant care, which can also lead to social isolation as they have less time for other relationships [1,4]. Additionally, dementia care can create a financial burden, especially with limited support services and financial aid available within the health care system [3,6]. This leaves caregivers shouldering the primary responsibility for their loved ones’ well-being, often with inadequate resources.

Furthermore, research highlights a lack of knowledge and the prevalence of negative attitudes among dementia caregivers, hindering their ability to provide optimal care [10]. To address these challenges, exploring the potential of mHealth apps to enhance caregiver knowledge, foster positive attitudes, and improve care practices is crucial [3,11]. This exploration holds significant implications for advancing dementia care and support for both people living with dementia and their caregivers [2,5,12,13,19].

Phase 1 of this study anticipates the successful adaptation and validation of questionnaires on KAP and burden levels among dementia caregivers in the Malay language. This adaptation will ensure cultural relevance and accessibility for the Malaysian population. While in Phase 2, the study aims to evaluate the effectiveness of the *DemensiaKITA* mHealth app. This study will rigorously evaluate the *DemensiaKITA* mHealth app to determine its efficacy in enhancing knowledge, fostering positive attitudes, and improving care practices among dementia caregivers in Malaysia. Ultimately, this evaluation aims to demonstrate the app’s potential to alleviate caregivers’ burden within the Malaysian context.

This study holds significant implications for advancing dementia care in Malaysia by providing culturally relevant and validated questionnaires for assessing KAP and burden levels among dementia caregivers. Furthermore, the rigorous evaluation of the *DemensiaKITA* mHealth app will generate evidence-based decision-making regarding its implementation and potential to support dementia caregivers in Malaysia. The study’s findings are expected to contribute to the development of effective strategies for improving KAP and reducing caregiver burden, ultimately enhancing the health and well-being of both people living with dementia and their caregivers in Malaysia and other countries.

In addition, the findings of this study have the potential to guide national policies in Malaysia by providing evidence for the effectiveness of mHealth apps in dementia care. This could lead to the integration of the *DemensiaKITA* app or similar mHealth solutions into national dementia care strategies. Additionally, the findings could inform resource allocation for caregiver support, emphasizing the need for accessible and culturally relevant resources.

In clinical practice, health care providers, particularly those working in geriatric care, could incorporate the *DemensiaKITA* app into routine care by recommending it to caregivers during consultations. This would provide caregivers with easy access to information and support, potentially improving their KAP in dementia care. Effective dementia care can help prevent costly complications, reduce hospitalizations, and delay the need for long-term care. This can lead to significant cost savings for the health care system.

In terms of community impact, the availability and promotion of the *DemensiaKITA* app could have a positive impact on the community by increasing awareness and support for dementia caregivers. This could lead to a greater understanding and acceptance of dementia, fostering a more dementia-friendly society in Malaysia. By supporting caregivers and improving their well-being, the app can enable them to remain in the workforce or continue with their daily activities, contributing to economic productivity.

It is anticipated that there will be several challenges while conducting this study. Recruiting and retaining participants for both phases of the study, particularly in Phase 2, may be challenging, especially considering the sensitive nature of dementia caregiving and the time commitment required for follow-up assessments. Moreover, adapting and validating the questionnaires into the Malay language requires careful consideration of cultural nuances to ensure the accuracy and relevance of the instruments for the Malaysian population. Additionally, the effectiveness of the *DemensiaKITA* app in improving caregiver KAP and reducing burden levels may be influenced by factors such as individual caregiver characteristics, the severity of dementia in the care recipient, and access to technology and support. Besides that, data collection and analysis can be time-consuming and resource-intensive, requiring careful planning and execution to ensure the accuracy and reliability of the results.

### Strengths and Limitations

This study acknowledges several limitations. First, the findings may not be fully generalizable to all dementia caregivers in Malaysia. The research focuses on caregivers attending geriatric clinics in Selangor and Kuala Lumpur, potentially excluding those in rural or underserved areas with differing needs and access to resources.

Second, the nonrandomized controlled trial design, while chosen due to anticipated challenges in recruitment and sample size, may introduce selection bias. This limitation could affect the generalizability of the findings to the broader population of dementia caregivers.

In addition, the app’s current availability only on the Android platform poses a significant limitation. This restricts access for a portion of caregivers who use iOS or other operating systems, potentially introducing further bias and limiting the study’s reach and impact.

Nevertheless, this study possesses several notable strengths. First, the *DemensiaKITA* mHealth app is uniquely positioned as a culturally and linguistically tailored solution for Malaysian dementia caregivers. This addresses a critical gap in existing mHealth apps, which often cater to English-speaking populations in high-income countries, leaving caregivers in Malaysia with limited access to relevant and accessible support tools. The app’s comprehensive design includes features aimed at enhancing knowledge, fostering positive attitudes, improving care practices, and reducing caregiver burden across multiple domains (emotional, physical, social, and financial), offering a holistic approach to dementia care support.

Second, the offline functionality is a major advantage in Malaysia, where internet access can be limited in rural areas. This ensures equitable access to support and resources for caregivers across the country. In addition, the app’s free availability removes financial barriers, making it accessible to all caregivers, regardless of their socioeconomic background. This promotes inclusivity and reduces health disparities.

Third, the study uses a robust, nonrandomized controlled trial design with follow-up assessments at baseline, 1, and 3 months, enabling a thorough evaluation of the intervention’s effectiveness over time. This longitudinal approach provides valuable insights into the app’s impact on caregiver outcomes and its potential for long-term benefits. Furthermore, the study’s comprehensive evaluation of knowledge, attitude, practice, and burden provides a holistic understanding of the intervention’s impact on caregivers’ well-being and their capacity to provide quality care to people living with dementia.

Fourth, by focusing on dementia caregivers in Malaysia, the study addresses a population facing a disproportionately high caregiver burden, reported at 69.4% [8]. This highlights the urgent need for effective interventions to support caregivers in this context and emphasizes the study’s potential to make a significant contribution to addressing this vital issue.

In addition, while the nonrandomized design and purposive sampling may introduce limitations, they also enhance the study’s practical relevance. By mirroring real-world settings in which interventions are often rolled out to specific clinics or populations, the study offers valuable insights into the feasibility and effectiveness of implementing the *DemensiaKITA* app within the existing health care infrastructure in Malaysia.

### Conclusion

This study protocol embarks on a crucial mission to improve the health and well-being of dementia caregivers in Malaysia. First, it focuses on adapting, translating, and validating questionnaires to assess their KAP and burden levels. In addition, this study is pioneering research on the effectiveness of the *DemensiaKITA* mHealth app, designed to support dementia caregivers in Malaysia through a nonrandomized controlled trial at baseline, 1-month follow-up, and 3-month follow-up.

As dementia becomes more prevalent, this app offers a crucial lifeline for caregivers facing increasing caregiving challenges. It is culturally tailored for Malaysia and works offline, making it relevant to countries with limited internet access. This research will provide valuable insights into the ways in which this technology can improve dementia care globally. Furthermore, the study will generate culturally relevant and validated questionnaires for researchers and health care professionals to accurately assess KAP and burden levels among dementia caregivers. These data can inform the development of targeted interventions and support programs.

Moreover, the findings of this study can contribute to evidence-based decision-making regarding the implementation of mHealth interventions in dementia care, paving the way for wider adoption and integration into national health care strategies. By enhancing caregiver KAP and reducing their burden, the *DemensiaKITA* app can empower caregivers to provide better care, cope with challenges, and maintain their well-being, leading to improved outcomes for people living with dementia and their caregivers.

In conclusion, this study protocol represents a significant step forward in dementia care research and practice in Malaysia. A rigorous and culturally sensitive approach promises valuable insights into effective strategies for supporting dementia caregivers and improving the lives of those affected by this debilitating disease. This research is a testament to the power of the mHealth app to address pressing health care challenges and promote the well-being of vulnerable populations.

## Abbreviations

CTI-25: Caregiver Task Inventory—25 items
CVI: content validity index
DAS: Dementia Attitudes Scale
DKAT-2: Dementia Knowledge Assessment Tool Version 2
EFA: exploratory factor analysis
FVI: face validity index
GEE: generalized estimating equations
KAP: knowledge, attitudes, and practices
mHealth: mobile health
MOH: Ministry of Health
NRCT: nonrandomized controlled trial
UiTM: Universiti Teknologi MARA
UMMC: Universiti Malaya Medical Center
ZBI-12: Short-form Zarit Burden Interview—12 items
